# Personalized Physical Activity Coaching: A Machine Learning Approach

**DOI:** 10.3390/s18020623

**Published:** 2018-02-19

**Authors:** Talko B. Dijkhuis, Frank J. Blaauw, Miriam W. van Ittersum, Hugo Velthuijsen, Marco Aiello

**Affiliations:** 1 Johann Bernoulli Institute for Mathematics and Computer Science, Faculty of Science and Engineering (FSE), University of Groningen, Nijenborgh 9, 9747 AG Groningen, The Netherlands; f.j.blaauw@rug.nl (F.J.B.); m.aiello@rug.nl (M.A.); 2 Institute of Communication, Hanze University of Applied Sciences, Media and ICT, Zernikeplein 11, 9746 AS Groningen, The Netherlands; h.velthuijsen@pl.hanze.nl; 3 Developmental Psychology, University of Groningen, Grote Kruisstraat 2/1, 9712 TS Groningen, The Netherlands; 4 School for Health Care Studies, Hanze University of Applied Sciences, Petrus Driessenstraat 3, 9714 CA Groningen, The Netherlands; m.w.van.ittersum@pl.hanze.nl

**Keywords:** physical activity, machine learning, coaching, sedentary lifestyle

## Abstract

Living a sedentary lifestyle is one of the major causes of numerous health problems. To encourage employees to lead a less sedentary life, the Hanze University started a health promotion program. One of the interventions in the program was the use of an activity tracker to record participants' daily step count. The daily step count served as input for a fortnightly coaching session. In this paper, we investigate the possibility of automating part of the coaching procedure on physical activity by providing personalized feedback throughout the day on a participant’s progress in achieving a personal step goal. The gathered step count data was used to train eight different machine learning algorithms to make hourly estimations of the probability of achieving a personalized, daily steps threshold. In 80% of the individual cases, the Random Forest algorithm was the best performing algorithm (mean accuracy = 0.93, range = 0.88–0.99, and mean F1-score = 0.90, range = 0.87–0.94). To demonstrate the practical usefulness of these models, we developed a proof-of-concept Web application that provides personalized feedback about whether a participant is expected to reach his or her daily threshold. We argue that the use of machine learning could become an invaluable asset in the process of automated personalized coaching. The individualized algorithms allow for predicting physical activity during the day and provides the possibility to intervene in time.

## 1. Introduction

Unhealthy lifestyles lead to increased premature mortality and are a risk factor for sustaining noncommunicable diseases (NCDs) such as cardiovascular diseases, cancers, chronic respiratory diseases, and diabetes [1]. NCDs caused 63% of all deaths that occurred globally in 2008 [1]. There are four behavioral factors that have a significant influence on the prevention of NDCs: healthy nutrition, not smoking, maintaining a healthy body weight, and sufficient physical activity. Insufficient physical activity is one of the leading risk factors for the major NCDs and not meeting the recommended level of physical activity is associated with approximately 5.3 million deaths that occurred globally in 2008 [2]. 

A high amount of sedentary time without sufficient daily physical activity leads to a higher rate of all-cause mortality [3]. Besides the increased risk of premature mortality in the long term, the short-term quality of life, being able to work, and social participation is also threatened by insufficient physical activity [4]. Fortunately, these risks are eliminated when this sedentary time is compensated for with sufficient physical activity of moderate intensity [3]. 

In Western civilization, living a sedentary lifestyle is the rule rather than the exception, as many people work in office environments. In pursuance of preventing the negative effects of insufficient physical activity in the workplace, the Hanze University of Applied Sciences Groningen (HUAS), a large university in the northern part of the Netherlands, started a novel initiative named (in Dutch): ‘Het Nieuwe Gezonde Werken’ (The New Healthy Way of Working; HNGW). With HNGW, the HUAS aims to promote a healthy lifestyle and physical activity during the workday. HNGW consists of providing participants with educational group meetings, food boxes with healthy recipes, and individual coaching sessions supplemented with an activity tracker. Despite the fact that participants are coached every two weeks and measured continuously, it remains difficult for a coach to provide timely personalized feedback. The manual task of creating personalized feedback is time consuming, and as such it is not always possible for the participants to get in-depth and timely daily feedback on their progression. Furthermore, current activity trackers do not provide a prediction for reaching the daily goal.

In order to fill this gap, we propose a novel, personalized, and flexible machine-learning-based procedure that can automate a part of the coaching process and serve as a source of information on a participant's progress with physical activity during the day. The personalized model provides, throughout the day, information on the probability of the participant meeting his or her daily physical activity goal. We demonstrate the accuracy and effectiveness of this solution in practice by training different machine learning algorithms and evaluating their performance using a train-test split dataset from the HNGW data. We apply techniques like grid search and cross-validation to optimize each model in order to find their best configuration. To show the applicability of this research in practice, we developed a proof of concept Web application, which has, to the best of our knowledge, not been done before. With the personalized actionable information the application provides, we demonstrate that machine learning automating is feasible as a part of the coaching process. The techniques described in this work could serve two goals in the field of personalized coaching. Firstly, we envision how coaches can use such applications and how these applications can provide them with detailed insight about the participants’ activity during the day. Secondly, the tool could be used as a self-support tool, in which the participants’ engagement with their lifestyle might increase as a result of the extra feedback.

## 2. Related Work

A number of studies have been performed on physical activity over days, where the sources of variance in activity is related to the subject, the day of the week, the season, and occupational and non-occupational days [5]. Tudor-Locke et al. (2005) showed that the individual is the main source of variability in physical activity next to the difference between the Sunday and the rest of the week [6]. Another study identified physical inactivity being lower on weekend days, and Saturday was the most active day of the week for both men and women [5].

To reduce sedentary time and increase physical activity levels, individuals need to change their behavior and daily routines. This is hard to achieve because of various reasons, and requires interventions and coaching strategies that use well-established techniques to induce a behavior change. A review by Gardner et al. (2016) found that self-monitoring, problem solving, and restructuring the social or physical environment were the most promising behavior change strategies, and—although the evidence base is quite weak—advises environmental restructuring, persuasion, and education to enhance self-regulatory skills [7]. Interventions aimed at increasing physical activity levels or reducing sedentary time varies widely in content and in effectiveness. For example, studies focusing on exercise training and behavioral approaches have demonstrated conflicting results, whereas interventions focusing on reducing sedentary time seem to be more promising [8,9,10,11,12]. The use of active video games seems to be effective in increasing physical activity, but has inconsistent findings on whether they are suitable to meet the recommended levels [13]. Also, interventions targeting recreational screen time reduction might be effective when using health promotion curricula or counseling [14]. Web- or app-based interventions to improve diet, physical activity, and sedentary behavior can be effective. Multi-component interventions appear to be more effective than stand-alone app interventions, although the optimal number and combination of app features and level of participant contact needed remain to be confirmed [15,16]. The workplace is often used for health promotion interventions. Recent reviews on workplace interventions for reducing sitting at work found initial evidence that the use of alternative workstations (sit-stand desks or treadmills) can decrease workplace sitting by thirty minutes to two hours. In addition, one review found that interventions promoting stair use and personalized behavioral interventions increase physical activity, while the other found no considerable or inconsistent effects of various interventions [17,18].

Step counters provide an objective measure of activity levels and enable self-monitoring. Furthermore, most modern consumer-based activity trackers already contain several behavior change models or theories [19,20]. Therefore, based on the aforementioned, using activity trackers in interventions to promote healthy lifestyles is promising. From meta-analyses by Qiu et al. and Stephenson et al. it was concluded that step counter use was indeed associated with small but significant effects in reducing sedentary time [21,22]. Adding an activity tracker to physical therapy or counseling was effective in some populations [23,24,25]. Besides collecting activity data for therapy or counseling, it is known that the Fitbit itself also serves as an intervention mechanism [26]. The mere fact of wearing an activity tracker (even without any form of coaching) could motivate physical activity and improve health-related quality of life [27,28]. On the other hand, studies on workplace interventions using activity trackers report conflicting results [29,30,31,32,33].

There are several studies that use sensor or activity tracker data to build a custom-made application to support research. An example is the social computer game, Fish'n'Steps, which connects the daily steps of an employee to the growth and activity of the individual avatar fish in a virtual fish tank. The more one is active, the faster the fish grows and prospers [34]. Another example is the study on increased physical activity as the effect of social support groups using pedometers and an app [35].

Although applying machine learning to coaching is new, machine learning techniques in combination with sensors have been applied before to identify the type of activity. Identifying human activity using machine learning and sensor data have been studied, for example, by Wang et al. for recognizing human daily activities from an accelerometer signal [36], by Li et al. on the quantification of the lifetime circadian rhythm of physical activity [37], or by Catal et al. on the use of an ensemble of classifiers for accelerometer-based activity recognition [38]. Only a few studies have investigated the use of actionable, data-driven predictive models. A study on creating a predictive physical fatigue model based on sensors identified relevant features for predicting physical fatigue, however the model was not proven to be predictive enough to be applied [39].

In order to improve physical activity in combination with activity trackers, a coaching feature is helpful, but only when the messages are personal and placed in context [40]. Perceiving the coaching information as personal and relevant is crucial for the effectiveness of (e)Coaching [41]. Such tailored (e)Coaching has many aspects, two of which are personalization and timing [42]. Timeliness of information is important for participants to be able to process the information and apply the advice while it is still relevant for them. In order to provide such advice, access to real-time predictions is vital, as it allows for timing the moment of coaching, either virtual or in real life and as flexible as needed. To the best of our knowledge, no studies exist about the use of sensor data combined with machine learning techniques for creating validated and individualized predictive models on physical activity. The individualized models could help the coach and the participant in the process of behavior change and increased physical activity.

## 3. Materials and Methods

The present work revolves around the HNGW project. This project was started in 2015 and focuses on promoting a healthy lifestyle. We describe the design of this study and how the resulting data is used in the present work. Next we describe our analysis pipeline. We describe the conversion of the raw data set into a feature set, the evaluation methods of the predictive models, and the choice of the algorithms. Finally we shed light on the proof of concept application we created to demonstrate how these techniques could be used in practice.

### 3.1. Study Design

The goal of the workplace health promotion intervention HNGW at the HUAS was to increase physical activity during workdays, by improving both physical and mental health, and several work-related variables. In the study, several performance-based tests and self-reported questionnaires were used to assess its effectiveness on a group level. 

Forty-eight eligible participants from the HUAS were randomized into two groups, stratified according to age, gender, BMI, and baseline self-reported health. One group followed a twelve-week workplace health promotion intervention; the other served as a control during the first twelve weeks and thereafter received the twelve-week workplace health promotion intervention.

During the study, minutely step count data of the participants was collected. Step count was measured using a wrist-worn activity tracker, the Fitbit Flex. The Fitbit Flex has been shown to be a reliable and valid device for step count and suitable for health enhancement programs [13]. Further details of the trial design on HNGW at the HUAS are represented in the manuscript of van Ittersum et al. [43].

### 3.2. Data Set

The anonymized data used in the present study was collected from participants during their participation in the HNGW health promotion program. All participants provided informed consent for participation in the HNGW study and for the use of their anonymized data for research purposes.

We used the steps per minute of each participant, resulting in a total of 349,920 measurements across all participants. We only considered the step data collected during the intervention period. That is, for both the intervention and the control group, we used the last twelve weeks of available step data. By focusing on the intervention period, we have a more homogeneous sample than we would have when including both the intervention and control data.

While the Fitbit platform provides us with several minutely measures (e.g., steps, metabolic equivalent of tasks [METs], calories, and distance), in our analysis we only included the steps variable. We used the steps variable as we expect it to be the most accurate and relevant, as all other variables are by-products derived using approximation algorithms.

### 3.3. Data Processing, Transformation, and Performance

To prepare the available minutely step data as input for training the algorithms, we first performed a data cleaning, reformatting, and pre-processing step. First, we removed incomplete days from the data set. We also removed all days with zero steps and weekend days. We then converted all provided variables in a format that could be used by our algorithms, by augmenting our initial data set with several new augmented variables, such as hour of the workday, the number of steps for that hour, and a cumulative sum of the number of steps till that hour. 

Note that we define a workday as the weekdays Monday to Friday. The normal working hours at the university are between 8:00 AM and 5:00 PM. The HNGW tried to motivate the participants to walk at least a part of the distance they commute daily. As a consequence, the hours of interest are the combination of the working hours and the period of commuting. Therefore we only considered the number of steps per hour between 7:00 AM and 6:00 PM. As features for training the algorithms, we used the hour per workday (ranged from 7:00 AM to 6:00 PM), the number of steps of that hour, and the cumulative sum of the number of steps till that hour.

As the outcome measure, we calculated the average number of steps for all workdays over all weeks. That is, for each individual, we calculated one average for all workdays. We considered the number of steps between 7:00 AM and 6:00 PM. Note that this outcome measure is not used as input in the training process. We constructed a binary outcome variable represented by the indicator variable $Y_{j}=Ι\left(s_{j}\geq \theta_{j}\right)$, in which $s_{j}$ refers to the number of steps on a workday for individual j, and $\theta_{j}\;$refers to the specific step goal for that j. The indicator function returns one (the ‘true’ label) when the inside condition holds, and zero (the ‘false’ label) otherwise.

Three days of repeated measures are necessary to represent adults’ usual activity levels with an 80% confidence [6]. Forty-four participants met the criteria. The processing and transformation for these forty-four participants resulted in a total of 120,480 data blocks (for the number of steps, mean = 9031, median = 8543, range = 0–47,121). The total number of positives when the threshold is met at 6:00 PM, is 1528. The total number of negatives when the threshold is not met at 6:00 PM, is 1879.

Note that we did not include any of the group level/baseline variables like age or gender, as we only considered personalized models. Although these variables might affect the outcome, they do not vary within the individual and as such do not add information.

### 3.4. Evaluation of the Performance of Algorithms and Models

We trained eight different machine learning algorithms. To compare their performance, we used a method known as ‘confusion matrices’. The confusion matrices give an overview of the true positives (TP; the model predicted a ‘true’ label and the actual data contained a ‘true’ label), true negatives (TN; the model predicted a ‘false’ label and the actual data turned out to have a ‘false’ label), false positives (FP; the model predicted a ‘true’ label, but the actual data contained a ‘false’ label), and false negatives (FN; the model predicted a ‘false’ label, but in fact the data contained a ‘true’ label) of a model. An example of a confusion matrix is provided in Table 1. These confusion matrices served as a basis for the calculation of two other performance measures: The accuracy and the F1-score [15].

Accuracy is a metric to determine the nearness of the prediction to the true value. A value of the accuracy close to one indicates the best performance. It calculates the ratio between the correctly classified cases and all cases as Accuracy = $\frac{TP+TN}{TP+TN+FP+FN}$.

Besides the accuracy metric, we calculated the F1-score for each model. Similar to the accuracy metric, the F1-score takes its values from between zero and one, one corresponding to the best performance. To calculate the F1-score, we use two other metrics known as the precision and the recall of the model. Precision is the proportion of the true positives and the false negatives, and is calculated as Precision = $\frac{TP}{TP+FN}$.

Recall is the true positive rate, which is calculated as Recall = $\frac{TP}{TP+FP}$.

Using these definitions of precision and recall, the F1-score can be calculated as F1-score = $2\times \frac{Precision\times Recall}{Precision+Recall}$.

### 3.5. Computing the Personalized Predictive Model

We aim to predict (throughout the day) whether or not an individual will meet his or her daily step goal. Prediction of meeting a set goal is a supervised two-class classification problem. Nowadays, many different algorithms for performing such classifications are available. Unfortunately, it is generally considered impossible to determine *a priori* which algorithm will perform best on any given data set [44]. Although distinct algorithms are better suited for different types of data and problems, the type of algorithm is merely an indication of the most suitable algorithm. Currently, the preferred way to find the best-performing algorithm is by empirically testing each of them [45]. Nevertheless, there exist general guidelines to direct the search for specific algorithms for the problem at hand. One of the leading organizations on open source machine learning library, scikit-learn.org, offers a flowchart about which algorithms can be chosen in which situation [46]. Also, Microsoft provides a ‘cheat sheet’ on their Azure machine learning platform [47]. The flow chart and ´cheat sheet´ served as a basis for our selection process and we chose the following machine learning classification algorithms: (i) AdaBoost (ADA), (ii) Decision Trees (DT), (iii) KNeighborsClassifier (KNN), (iv) Logistic Regression (LR), (v) Neural Networking(NN), (vi) Stochastic Gradient Descent (SGD), (vii) Random Forest (RF), and (viii) Support Vector Classification (SVC). The performance of each of these algorithms was first determined for seventy percent of the whole dataset including five-fold cross-validation with scaling of the factors for KNN, NN, SGD, and SVC. Subsequently, for every participant we individualized the algorithms with five-fold cross-validation and grid search on selected hyperparameters. Seventy percent of the available individual data was used as training data. After training the algorithms, the algorithms were turned into persistent predictive models per participant. We used the individual models to construct confusion matrices, which in turn served as a basis for the F1-score and the accuracy per individual predictive model. To compare the performance of the machine learning models, we included a baseline model. This baseline model checks the cumulative step count. If this cumulative step count equals or exceeds the average personalized goal, the model returns true and false otherwise. We ranked all machine learning models (including the baseline model) using the average of the F1-score and the accuracy.

### 3.6. Proof of Concept

We designed and implemented a Web application to demonstrate how the personalized prediction based on machine learning and activity tracker data could be used in practice. We developed this application as a Web application, which can be accessed on http://personalized-coaching.compsy.nl/. In this application, the user can input the values ‘Hour of the day’, ‘Steps previous hour’, ‘Total steps till the Hour’, combined with the participant’s ID and the algorithm to use. The Web application then uses the individualized model and input data to predict the outcome together with the probability thereof.

### 3.7. Implementation Details

We used scikit-learn (v0.18, [48]) to establish the best predictive model for the individual. Scikit-learn is an open-source Python module integrating a wide range of machine learning algorithms. Scikit learn was integrated in Anaconda (v4.2.13, [49]) and Jupyter Notebooks (4.0.6, [50]) was used in combination with Python (v3.5.2, [49]) for creating the data processing and machine learning pipeline. Jupyter Notebooks is an interactive method to write and run various programming languages, such as Python. The participants, their physical activity data, and the results of the performance of the algorithms and models were saved in an Oracle database (v11g2 XE; [51]). The Oracle database management system is a widely-used SQL-based system for persisting data. The source code and corresponding notebooks of the machine learning procedure is available as open-source software on Github (https://github.com/compsy/personalized-coaching-ml).

For the Web application, we used Flask (Version 0.10.1, [52]), a Python-based Web application microframework for developing Web applications. We used a PostgreSQL database to store information regarding the models and the participants. The machine learning models resulting from the pipeline are exported as Python Pickle files, which were imported into the Web application. The infrastructure-as-a-service provider Heroku is used to host a demo version of the Web application. This Web application is available at http://personalized-coaching.compsy.nl. The Web application is available as open-source software on Github (https://github.com/compsy/personalized-coaching-app).

## 4. Results

After optimizing our machine learning models by applying grid search in combination with cross-validation, we assessed the models using the test set. The results are presented here.

### 4.1. Accuracy and F1-Score on Group Level

Table 2 presents the F1-score and accuracy of the eight different algorithms at the group level. The top three group algorithms based on the mean accuracy and F1-score are: 

AdaBoost, Neural Networking, and Support Vector Classifier.

**Figure 1:**
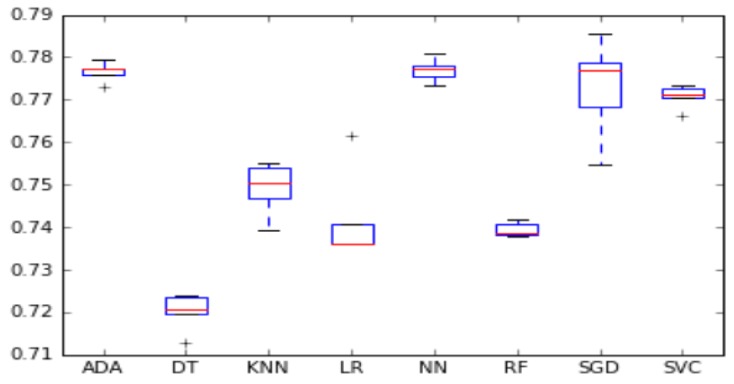
Algorithm accuracy comparison.

**Figure 2:**
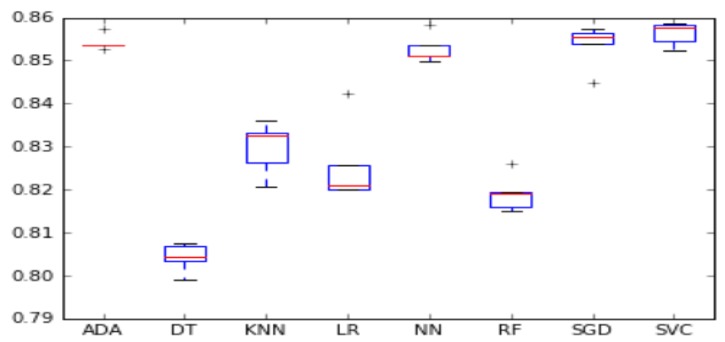
Algorithm F1-score comparison.

We visualized the accuracy and F1-score per algorithm using boxplots in Figure 1 and Figure 2. The box represents the second and third quartile groups and the red line indicates the median. The upper whisker visualizes the fourth quartile group and the lower whisker visualizes the first quartile group. Finally, the plus sign indicates outliers on either side of both whiskers.

### 4.2. Individual Algorithms

We trained all algorithms on the training set of each individual and performed cross-validation to tune the hyperparameters. Table 3 lists the used machine learning algorithms, the set of tested hyperparameters, and the selected grid search values.

**Figure 3:**
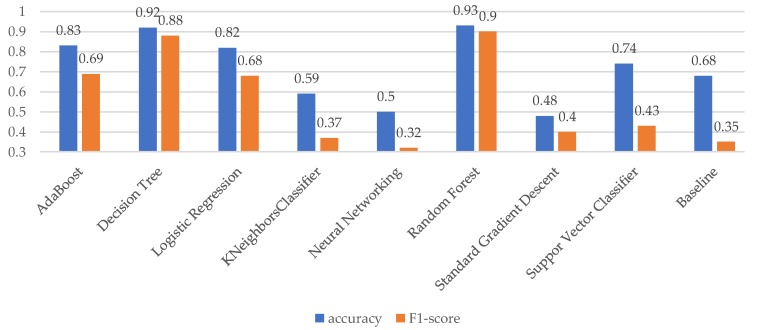
Average accuracy and F1-score per model.

The accuracy and F1-score of the individual algorithms differ. Figure 3 visualizes the results of the average of the individual scores.

For thirty-five subjects, the best-performing individual model was the Random Forest algorithm, in eight cases this was the Decision Tree algorithm, and for one subject the AdaBoost algorithm performed best. The average accuracy of the Random Forest algorithm is 0.93 (range 0.88–0.99). Thus, in terms of accuracy, the individual Random Forest models score better than its counterpart that was generalized over all individuals (mean personalized accuracy = 0.93 versus mean generalized accuracy = 0.82). The average accuracy of the Decision Tree model is 0.93 (range 0.91–0.97) and outperforms the generalized, group-based Decision Tree accuracy of 0.75. The accuracy of the single AdaBoost model is 0.98, which outperforms the group accuracy of 0.85.

The mean F1-score of the Random Forest model is 0.90 (range 0.87–0.94). The mean F1-score of the Decision Tree model based on the eight best performing models is 0.90 (range 0.87–0.93). Finally, the best AdaBoost model has an F1-score of 0.92, while the group accuracy for the AdaBoost algorithm was 0.77.

The use of grid search to tune the hyperparameters of the algorithms led to several optimized models per individual. To demonstrate the difference this optimization operation can have, we present an example of two individual models with different hyperparameter configurations in Table 4. Table 5 gives an overview of the number of occurrences of a value for the Random Forest hyperparameters. 

**Figure 4:**
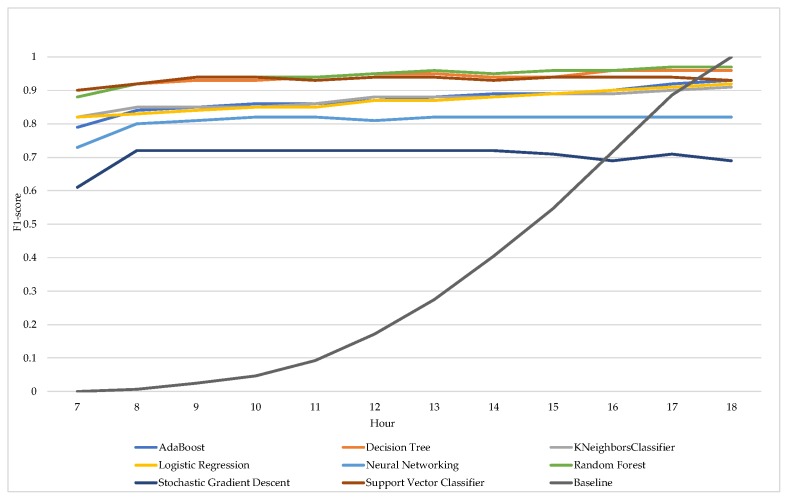
Average F1-Score per algorithm, per hour based on the individual scores.

**Figure 5:**
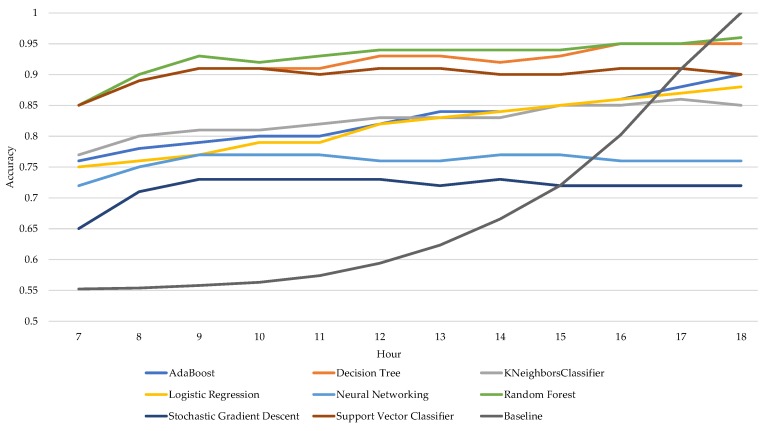
Average accuracy per algorithm, per hour based on the individual scores.

The accuracy and F1-score of the various machine learning algorithms increase slightly during the day. The size of this increase differs slightly per machine learning algorithm. For instance, the F1-score of Random Forest increases with 10% during the day, starting with an F1-score of 0.89 at 7:00 AM and ending with an F1-score of 0.97 at 6:00 PM. Both Figure 4 and Figure 5 also show the increase in accuracy and F1-score of the baseline algorithm during the day. Its accuracy starts with 0.55 and ends at 1 at the end of the workday, while the F1-score starts at 0 and ends at 1. The accuracy increases for Random Forest, Logistic Regression, and AdaBoost, whereas the accuracy of Neural Networking is best at 11:00 AM and Stochastic Gradient Descent remains the same.

### 4.3. The Web Application

**Figure 6:**
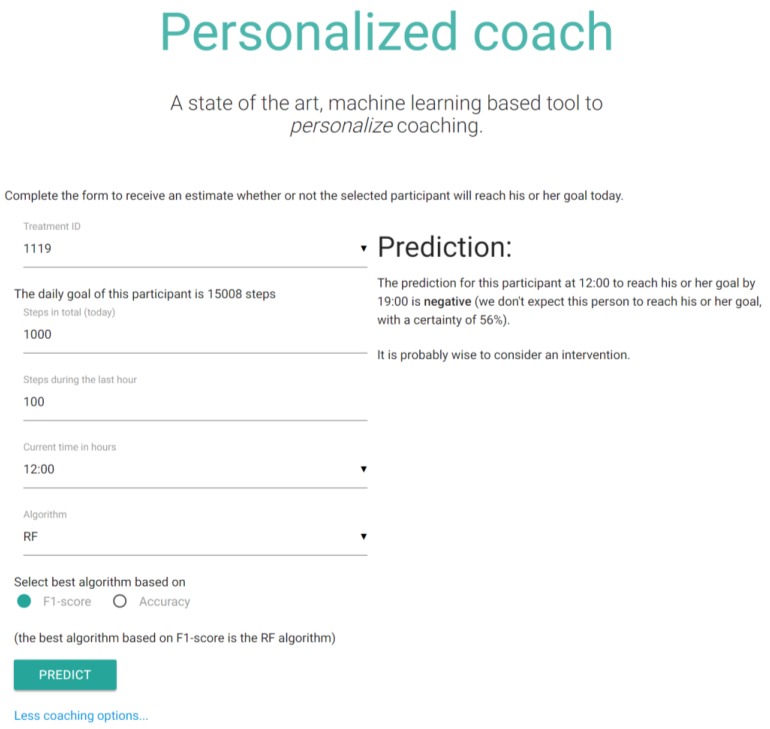
Screenshot of the Personalized Coach Web Application.

The Web application is a demonstration of how the aforementioned machine learning techniques could be used in practice, from the perspective of both the coach and the participant. The application allows the user to determine whether a participant will achieve his or her goal for the day, during the day, by applying the individualized algorithms. The procedure for predicting this goal is as follows. First, the user selects a participant identifier from the dropdown menu. After this selection had been made, the application selects the best and personalized machine learning algorithm for this specific participant. Then the user can fill out a form, providing the necessary details to base the prediction on (hour of day, the number of steps so far, and the number of steps in the past hour). Finally, when the user submits the form, the application returns advice personalized for the individual selected from the dropdown menu. The demo application is available at http://personalized-coaching.compsy.nl/. Figure 6 provides a screenshot of both the input fields of the application and the generated prediction and advice.

## 5. Discussion

We investigated machine learning as a means to support personalized coaching on physical activity. We demonstrated that for our particular data sets, the tree algorithms and tree-based ensemble algorithms performed especially well. To demonstrate how the results of machine learning techniques could be used in practice, an application was used to aid the coaching of the physical activity process. Furthermore, the analysis shows that selecting the right algorithm, using the dataset of the individual participant, and tuning its individual algorithm parameters, can lead to significant improvements in predictive performance and is a critical step in machine learning application. All source code, including the different notebooks and the proof-of-concept Web application is available online as open-source software. The source code can serve as a blueprint for other researchers when aiming to apply machine learning for coaching. 

Although Random Forest outperformed most of the other algorithms, it is problematic to provide a generalized recommendation for specific algorithms, parameters, or parameter settings [44]. Presumably due to individually different physical activity patterns, different algorithms and parameters have to be considered. As a starting point, we selected the algorithms based on well-established sources [41,42], applied cross-validation, and grid-searched the values of the selected parameters. Nevertheless, it’s important to note that these algorithms, parameters, and grid search values might not work best on all individual physical patterns, and the algorithms, parameters, and grid search values should only be used as starting points. Future work might consist of investigating the underlying mechanisms to be able to choose the best algorithm beforehand.

We based the prediction solely on the hour of the day and the number of steps. These steps are naturally increasing over the day, and as such, not independent from each other. By including the cumulative number of steps for each block of data, and by including the number of steps made in the past hour, we assume each block to be independent from the other blocks, and as such, are still able to use the regular machine learning methods. 

A limitation of the present work is that all participants included in this study participated in an intervention. This intervention might have made the participants more aware and engaged with the project, and as such, the individualized models might be biased towards the best scenario. When people are not extrinsically motivated to meet their daily physical activity goal, and lower their physical activity, the predictive power of the models and therefor the effect of automated intervention will lessen. On the other hand, when an intervention like the health promotion program ends, the individualized models check the participant on his or her performance as if the program is supporting the participant.

As presented in the state of the art literature, the total number of steps differ significantly between Sunday and rest of the weekdays [5,6,48]. Within this health promotion program, the focus was on improving physical activity during working hours and commuting. Therefore, the machine learning models were trained based on the normal workweek. Only one model per participant, based on the five weekdays, is adequate to predict whether or not a participant will meet his or her threshold. It may be necessary to conduct different models for the weekend and weekdays when a health promotion program is expanded to weekends. A reason to establish more than one or two models per participant is found in the variances between weekdays [5]. Examples of different factors that could influence the level of physical activity are weekly sport obligations, weekly meetings, or lunch walks on certain days. Constructing a model per weekday might establish an even more personalized and precise prediction.

In the present work, we only train our machine learning algorithms on variables provided by the activity tracker, extending this set of variables with other (exogenous) variables from other data sources. For example, the data can be extended to include information on the changes in the weather conditions and/or season, which are known to correlate with the day-to-day activity [5,53], or non-working time during weekdays like national holidays and free time, or part-time working schedule, for the activity level differs between non-occupational and occupational time, or the influence and effectiveness of coaching and interventions. Adding the mentioned factors to the dataset might improve the predictive accuracy of the model and might increase the effectiveness of the coaching process.

To apply the personalized machine learning models effectively, they have to become a part of a larger ecosystem. An ideal coaching process is fully tailored to the individual participant. One of the most important characteristics of the personalization of a coaching strategy consists in the timing and ease to execute triggers to change behavior [54]. To support these two aspects of coaching, timely information on the participant and the effectiveness of the coaching strategy are needed. Coaching might not be limited to a personal real life coach but also may include virtual coaching. An example of a possible use of the system is: at the moment the participant doesn’t score a ‘yes’ for two hours in a row on the prediction of meeting his threshold, a notification is sent out to both the participant and the coach. On the basis of this notification, the participant and the coach can take action; the coach can timely intervene to stimulate his client to become physically active and the participant can become instantly more active. Blok et al. proposed a system which combines the real-time analysis of activity tracker data and other personal streaming data as well as the evaluation of virtual coaching strategies, which enables it to tune the coaching to the person [55]. The present work could serve as a central component of a virtual coach system like that envisioned by Blok et al. [55].

To make the information even more personal and relevant, a promising direction for future work is to include a prediction of the actual number of steps at the end of the day. Adding more (and personalized) information might strengthen the effectiveness of the system. To do so, we could apply a similar procedure to the one presented in this study, but instead replace our classification algorithms with regression machine learning algorithms. The predicted number of steps could be a valuable extension in addition to the currently implemented classification of the step goal.

To conclude, machine learning is a viable asset to automate personalized daily physical activity prediction. Coaching can provide accurate and timely information on the participants’ physical activity, even early in the day. This is the result of applying machine learning to the behavior of the individual participant as precisely and frequently measured by wearable sensors. The prediction of the participant meeting his goal in combination with the probability of such achievement allows for early intervention and can be used to provide support for personalized coaching. Also, the motivation for self-coaching might be increased, while every model is personalized and the results are better fitted to the situation. Furthermore, machine learning techniques empower automated coaching and personalization.

## Figures and Tables

**Table 1:** Confusion matrix.

		***True Class***	
		**Yes**	**No**
*Predicted class*	**Yes**	True Positives (TP)	False Negatives (FN)
	**No**	False Positives (FP)	True Negatives (TN)

True Positive: the threshold of daily steps was met and predicted; True Negative: the threshold of daily steps was not met and predicted; False Negative: the threshold of daily steps was met and not predicted; False Positive: the threshold of daily steps was not met and not predicted.

**Table 2:** Algorithms and their scores for the whole dataset.

Algorithm Name	Mean Accuracy (Standard Deviation)	Mean F1 (Standard Deviation)	Rank
AdaBoost (ADA)	0.776623 (0.002080)	0.854157 (0.001626)	1
Neural Networking (NN)	0.777774 (0.001545)	0.852797 (0.002938)	2
Support Vector Classifier (SVC)	0.770728 (0.002505)	0.856341 (0.002405)	3
Stochastic Gradient Descent (SGD)	0.767623 (0.005490)	0.853575 (0.004574)	4
KNeighborsClassifier (KNN)	0.749171 (0.005683)	0.829826 (0.005544)	5
Logistic Regression (LR)	0.742125 (0.009821)	0.825725 (0.008487)	6
Random Forest (RF)	0.737451 (0.003210)	0.819065 (0.003840)	7
Decision Tree (DT)	0.720535 (0.004787)	0.804220 (0.003006)	8

**Table 3:** Algorithms, used parameters, and grid search values.

Algorithm name	Hyperparameters	Values
AdaBoost (ADA)	n_estimators: number of decision trees in the ensemble	[10,50]
	learning rate: the shrink of the contribution of each successive decision tree in the ensemble	[0.1, 0.5, 1.0, 10.0]
Decision Tree (DT)	criterion: the algorithm to use to decide on split	[‘gini’, ‘entropy’]
	max_features: the number of features to consider when to split	[‘auto’,‘sqrt’,‘log2’]
KNeighborsClassifier (KNN)	metrics: the distance metric to use	[‘minkowski’,‘euclidean’,‘manhattan’]
	weights: weight function used	[‘uniform’,‘distance’]
	n_neighbors: number of neighbors to use for queries	[5, 6, 7, 8, 9]
Neural Networking (NN)	learning_rate_init: the control of the step-size in updating the weights	[‘constant’, ‘invscaling’, ‘adaptive’]
	activation: the activation function for the hidden layer	[‘identity’, ‘logistic’, ‘tanh’, ‘relu’]
	learning_rate: the rate for the weight of the updates	[0.01, 0.05, 0.1, 0.5, 1.0]
Logistic Regression (LR)	C: regularization strength	[0.001, 0.01, 0.1, 1, 10, 100, 1000]
	penalty: whether to use Lasso (L1) or Ridge (L2) regularization	[‘l1’, ‘l2’]
	fit_intercept: whether or not to compute the intercept of the linear classifier	[True, False]
Stochastic Gradient Descent (SGD)	fit_intercept: whether or not the intercept should be computed	[True, False]
	l1_ratio: the penalty is set to L1 or L2	[0,0.15,1]
	loss: quantification of the loss	[‘log’,‘modified_huber’]
Support Vector Classifier (SVM)	kernel: the kernel type to be used in the algorithm	[‘linear’,‘rbf’]
Random Forest (RF)	n_estimators:number of decision trees	[10, 50, 100, 500]
	max_features: the number of features to consider when to split	[0.1, 0.25, 0.5, 0.75, ‘sqrt’, ‘log2’, None]
	criterion: which algorithm should be used to decide on split	[‘gini’, ‘entropy’]

**Table 4:** Example of different tuned personalized Random Forest models.

Participant	Parameters	Values
1119	criterion max_features n_estimators	gini sqrt 50
1121	criterion max_features n_estimators	entropy log2 50

**Table 5:** The number of different values per Random Forest hyperparameter.

Hyperparameter	Value	Number of Occurrences
criterion	entropy	7
	gini	37
max_features	0.1	4
	0.25	5
	0.5	7
	0.75	15
	log2	2
	sqrt	2
	null	9
n_estimators	10	3
	100	17
	50	16
	500	6
